# Liquid Biopsy-Driven Cetuximab Rechallenge Strategy in Molecularly Selected Metastatic Colorectal Cancer Patients

**DOI:** 10.3389/fonc.2022.852583

**Published:** 2022-04-21

**Authors:** Stefano Mariani, Marco Puzzoni, Riccardo Giampieri, Pina Ziranu, Valeria Pusceddu, Clelia Donisi, Mara Persano, Giovanna Pinna, Erika Cimbro, Alissa Parrino, Dario Spanu, Andrea Pretta, Eleonora Lai, Nicole Liscia, Alessio Lupi, Enrica Giglio, Grazia Palomba, Milena Casula, Marina Pisano, Giuseppe Palmieri, Mario Scartozzi

**Affiliations:** ^1^ Azienda Ospedaliera Universitaria (AOU) Cagliari, University Hospital and University of Cagliari, Cagliari, Italy; ^2^ Medical Oncology Unit, University Hospital and Università Politecnica delle Marche, Ancona, Italy; ^3^ Unit of Cancer Genetics and Institute of Biomolecular Chemistry (ICB), National Research Council (CNR), Sassari, Italy

**Keywords:** colorectal (colon) cancer, epidermal growth factor receptor (EGFR), RAS, liquid biopsy, rechallenge, cetuximab

## Abstract

**Background:**

Rechallenge with EGFR inhibitors represents a promising strategy for patients with RAS wild type (WT) colorectal cancer (CRC) but definitive selection criteria are lacking. Recently, the RAS WT status on circulating tumor DNA (ct-DNA) emerged as a potential watershed for this strategy. Our study explored the liquid biopsy-driven cetuximab rechallenge in a RAS and BRAF WT selected population.

**Methods:**

CRC patients with RAS and BRAF WT both on tumor tissue and on ct-DNA at baseline receiving rechallenge with cetuximab were eligible for our analysis. Ct-DNA was analyzed for RAS-BRAF mutations with pyro-sequencing and nucleotide sequencing assays. Real-time PCR and droplet digital PCR were performed to confirm the RAS-BRAF mutational status.

**Results:**

A total of 26 patients were included in our analysis. In the global population, RR was 25.0%, median overall survival (mOS) was 5.0 months, and median progression-free survival (mPFS) was 3.5 months. Previous response to anti-EGFR was associated with improved mPFS (5.0 vs. 2.0 months, HR: 0.26, *p* = 0.048); anti-EGFR free interval > 14 months and anti-EGFR free interval > 16 months were associated with improved mPFS (respectively 7.0 vs. 3.0 months, HR: 0.27, *p* = 0.013 and not reached vs. 3.0 months, HR: 0.20, *p* = 0.002) and with improved mOS (respectively 13.0 vs. 5.0 months, HR: 0.27, *p* = 0.013 and 13.0 vs. 5.0 months, HR: 0.20, *p* = 0.002). Previous lines >2 were correlated with improved mPFS (4.0 vs. 1.0 month, HR: 0.05, *p* = 0.041) and with improved mOS (7.0 vs. 1.0 month, HR: 0.045, *p* = 0.034). In a multiple logistic regression model, only the anti-EGFR free interval was confirmed to be a significant predictor for mOS and mPFS.

**Conclusions:**

Liquid biopsy-driven cetuximab rechallenge was confirmed to be effective. The clinical outcome was consistent with available results from phase II studies. In addition to the molecular selection through the analysis of ct-DNA for RAS, the long anti-EGFR free interval is confirmed as a prospective selection criterion for this therapeutic option.

## Background

The anti-epidermal growth factor receptor (EGFR) antibodies (i.e., cetuximab and panitumumab) have a key role in the treatment of metastatic colorectal cancer. The resistance to anti-EGFR antibodies represents a challenging issue in this context. In particular the genetic heterogeneity of colorectal cancer along with the dynamic nature of tumor biology are at the basis of secondary resistance to EGFR blockade (1). The mechanism underlying the secondary resistance to EGFR blockade, although not yet fully understood, seems to be linked to the emergence of EGFR downstream pathway mutation including RAS mutations (2) and EGFR mutations (3). The K- and N-RAS mutations, in this respect, are the most important mediators of secondary resistance to EGFR blockade, and they can be detected by circulating cell-free tumor DNA analysis (2). The RAS and EGFR mutant alleles emerging at the time of disease progression to EGFR blockade have been shown to decline in blood during EGFR blockade suspension (4, 5). This biological phenomenon can be predicted by an exponential decay model (4, 5).

The rechallenge strategy with anti-EGFR might then represent a promising therapeutic weapon aiming to overcome secondary resistance to anti-EGFR in the light of the knowledge about the pulsatile behavior of RAS clones under EGFR blockade pressure. The basic idea supporting the concept of rechallenge with anti-EGFR is the possibility to successfully treat patients previously exposed and resistant to such drugs after an anti-EGFR interval in which tumor cells may have gone back to prevalent RAS wild-type (WT) status after developing a prevalent RAS mutant status due to anti-EGFR pressure.

Santini et al. (6) firstly investigated the activity of rechallenge with a cetuximab-based therapy in metastatic colorectal cancer patients achieving encouraging results in terms of clinical outcome. Response rate (RR) was 53.8% and median progression-free survival (mPFS) was 6.6 months.

Subsequently, Cremolini et al. (7) prospectively assessed the activity of cetuximab plus irinotecan as third-line treatment for patients with strict clinical criteria (i.e., prior first-line irinotecan and cetuximab-based regimen with at least partial response, progression-free survival of at least 6 months with first-line therapy, progression within 4 weeks after last dose of cetuximab, and prior second-line oxaliplatin and bevacizumab-based treatment). In the global population, RR was 21%, median PFS was 3.4 months, and median overall survival (OS) was 9.8 months. Particularly noteworthy was the finding of improved mPFS for patients with RAS wild-type ct-DNA (4.0 vs. 1.9 months, *p* = 0.03).

Similarly, Masuishi et al. (8) conducted a phase II trial of irinotecan-cetuximab rechallenge as third line confirming the clinical activity of this treatment strategy (3-month PFS rate was 44.1%, mPFS was 2.4 months, and mOS was 8.2 months). The long anti-EGFR free interval (>372 days) was related to an improved outcome in terms of RR, OS, and PFS.

Results from CHRONOS, a multicenter phase II trial of liquid biopsy-driven anti-EGFR rechallenge with panitumumab (9), have been presented at the ASCO 2021 annual meeting. Molecular inclusion criteria for enrolment in this study were very restrictive (patients showing a >50% drop in RAS mutational load compared to baseline were considered molecularly eligible). The study met the primary endpoint; overall response rate (ORR) was 30%, mPFS was 16 weeks, and, interestingly, response occurred independently of number of prior treatments and sidedness. Furthermore, the presence of resistance conferring mutations and responses were independent of the time since last anti-EGFR.

However, selection criteria allowing clinicians to reliably select patients most likely to benefit from such a treatment approach are yet to be defined. This is of particular clinical relevance when we consider that metastatic colorectal cancer patients who are potential candidates for a rechallenge with anti-EGFR may also be eligible, at the same time, for other therapeutic options with higher level of evidence (10, 11). In this scenario, liquid biopsy for RAS and BRAF mutational status should be considered a valuable criteria for patient selection (7, 9), as well as the hypothesis of delaying the introduction of rechallenge with anti-EGFR in order to both expose the patient to treatment options with phase III evidence (10, 11) of activity and take advantage of a longer anti-EGFR free interval.

Further studies are urgently needed to better understand the prognostic and predictive factors along with the better timeline for rechallenge strategy. The present study explored the role of liquid biopsy-driven rechallenge strategy with EGFR blockade in molecularly and clinically selected metastatic colorectal cancer patients.

## Methods

We conducted a multicenter retrospective analysis in patients with molecularly selected colorectal cancer who underwent rechallenge with anti-EGFR antibodies. The aim of the study was to evaluate the correlation of several putative predictive/prognostic factors for rechallenge strategy with clinical outcome. Patients were included in our analysis if they met the following criteria: histologically confirmed RAS and BRAF wild-type colorectal cancer, first-line anti-EGFR-based therapy with documentation of progression to first-line therapy within 4 weeks after the last administration of anti-EGFR, rechallenge with anti-EGFR antibody (e.g., irinotecan + cetuximab or cetuximab monotherapy), and measurable disease according to Response Evaluation Criteria in Solid Tumors (RECIST) version 1.1. To be eligible, patients must also have circulating tumor DNA (ct-DNA) RAS-BRAF WT profile at the rechallenge baseline. The ct-DNA was analyzed at the rechallenge baseline for RAS-BRAF mutations using pyro-sequencing (PyroMark Q24 MDx Workstation) and nucleotide sequencing (Genetic Analyzer ABI3130) assays. Real-time PCR (Idylla) and droplet digital PCR (QX200 System) were performed to confirm the RAS-BRAF mutation status. The limit of detection for RAS BRAF mutations was 5%. Tumor response was evaluated by clinicians’ assessment according to the Response Evaluation Criteria in Solid Tumors (RECIST v1.1). Several clinical variables including previous response to anti-EGFR containing therapy, anti-EGFR free interval, and previous lines for metastatic disease > 2 were evaluated in relation to outcome in terms of RR, mPFS, and mOS. Statistical analysis was performed with the MedCalc Statistical Software version 14.10.2 (MedCalc Software bvba, Ostend, Belgium; http://www.medcalc.org; 2014). For study purposes, the optimal anti-EGFR free interval threshold was defined according to receiver operating characteristic (ROC) curve analysis. The association between categorical variables was estimated by Fisher exact test for categorical binomial variables. Survival probability over time was estimated by the Kaplan–Meier method. Significant differences in the probability of survival between the strata were evaluated by log-rank test. Multiple logistic regression was used to assess the role of significant variables in the univariate analysis. The study was performed in accordance with the protocol, which was approved by the AOU Cagliari Ethical committee (approval number: 3.32 n.14 28/04/21) along with all experimental procedures. Written informed consent was obtained for all patients enrolled into the analysis, and methods were carried out in accordance with the Declaration of Helsinki.

## Results

A total of 26 patients with RAS-BRAF wild-type metastatic colorectal cancer receiving rechallenge with cetuximab between July 2018 and February 2021 were included in our analysis. All patients had RAS-BRAF wild-type status on ct-DNA at baseline. Overall clinical and pathological characteristics are summarized in **Table 1**. The median follow-up duration was 6.0 months (95% CI for the median 4.1 to 11.3). The most common treatment-related adverse events of any grade were skin rash (81%), diarrhea (35%), neutropenia (19%), and fatigue (12%). The most common grade 3 adverse event was neutropenia (8%). No grade 4 adverse events occurred.

**Table 1:** Overall clinical and pathological characteristics.

Number of patients	26		
Gender	Male	20 (76.9%)	
	Female	6 (23.1%)	
Median age	67 years	(95% CI: 62.5–73.0)	
ECOG Performance Status	0	8 (30.7%)	
	1	10 (38.5%)	
	2	8 (30.7%)	
RAS/BRAF WT status on tissue sample at diagnosis	100%		
RAS/BRAF WT status on ct DNA at baseline	100%		
Site of primary tumor	Left sided	25	(96.1%)
	Right sided	1	(3.8%)
Prior resection of the primary tumor	Yes	8	(30.8%)
	No	18	(69.2%)
Metastatic sites	Liver limited	7	(26.92%)
	Lung limited	4	(15.38%)
	Multivisceral	15	(57.7%)
Previous lines for metastatic disease	2	2	(7.7%)
	3	13	(50%)
	4	11	(42.3%)
First-line treatment	Oxaliplatin based	14	(60.9%)
	Irinotecan based	8	(30.8%)
	Cetuximab	8	(30.8%)
	Panitumumab	18	(69.2%)
Best response to first-line treatment	PD	5	(19.2%)
	RC	0	(0%)
	RP	17	(65.4%)
	SD	4	(15.4%)
Second-line treatment	Oxaliplatin based	4	(15.4%)
	Irinotecan based	19	(73.1%)
	Bevacizumab	12	(52.2%)
	Aflibercept	9	(39.1%)
Best response to second-line treatment	PD	6	(23%)
	RC	0	(0%)
	RP	10	(38.5%)
	SD	10	(38.5%)
Rechallenge regimen	Irinotecan + cetuximab	19	(73.1%)
	Cetuximab	7	(26.9%)
Median anti-EGFR free interval	11.5 months	(95% CI: 9.5–16.0)	
Response rate	25.0%		
Median overall survival	5.0 months	(95% CI: 4.0–11.7 months)	
Median progression-free survival	3.5 months	(95% CI: 2.5–6.0 months)	

**Figure 1:**
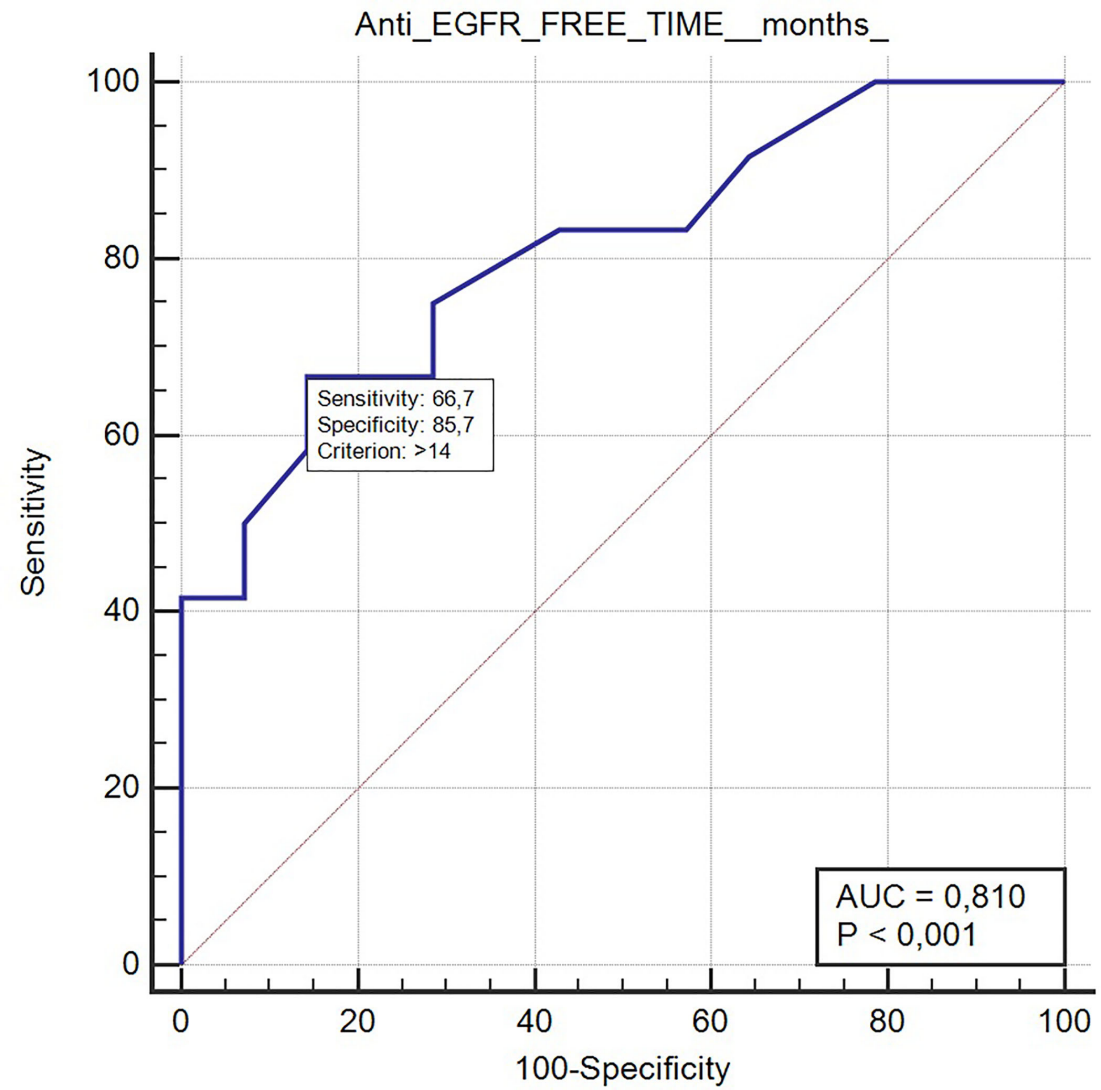
ROC curve of anti-EGFR free-time predictive of prognosis with rechallenge treatment (AUC 0.81, *p* < 0.001).

**Figure 2:**
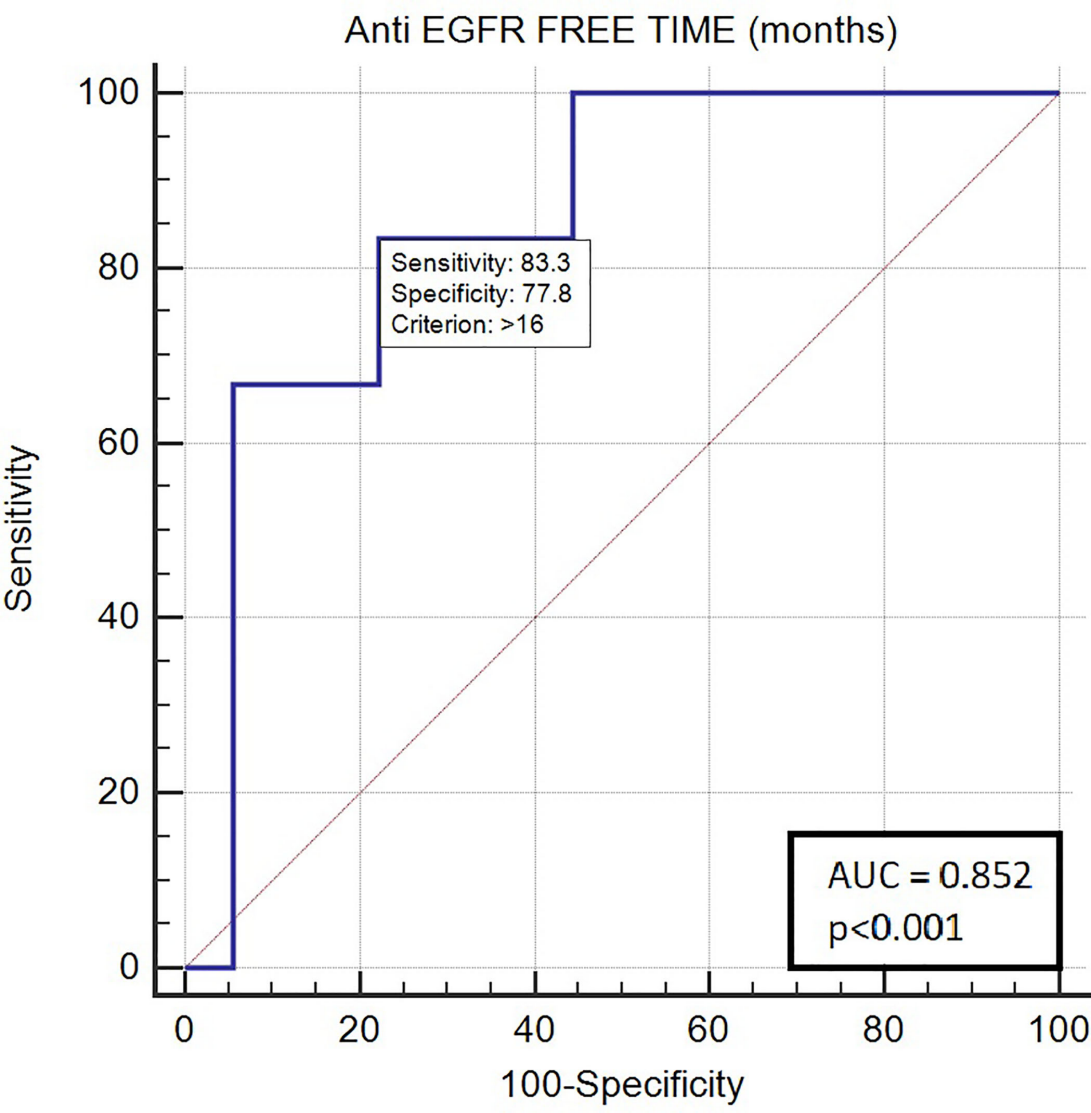
ROC curve of anti-EGFR free-time predictive of response with rechallenge treatment (AUC 0.852; *p* < 0.001).

**Figure 3:**
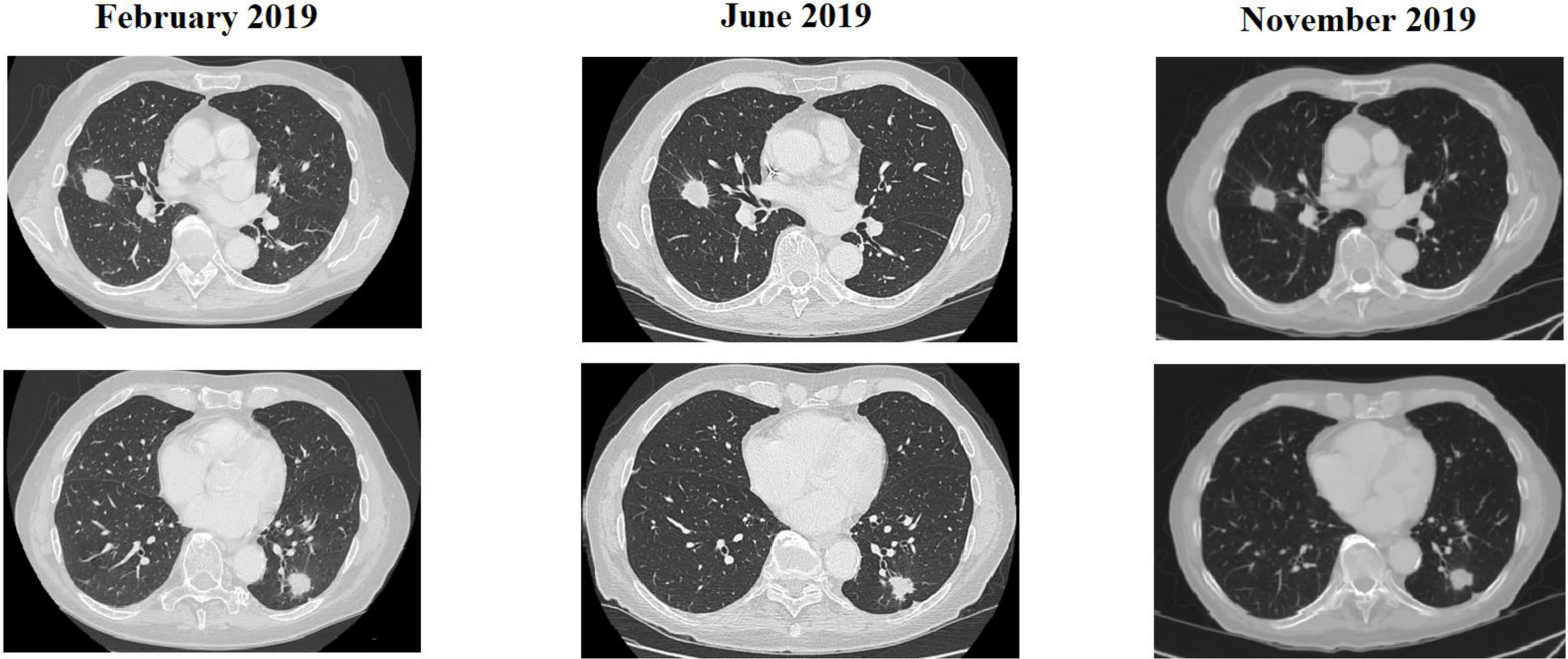
CT scan of a patient treated with rechallenge strategy after >2 prior lines of treatment and an anti-EGFR free interval > 16 months.

Long anti-EGFR free interval, previous response to anti-EGFR therapy, and previous lines >2 for metastatic disease were associated with clinical outcome in the univariate analysis. In particular, the previous response to anti-EGFR was associated with improved mPFS (5.0 vs. 2.0 months, HR: 0.26, *p* = 0.048); long anti-EGFR free interval > 14 months and long anti-EGFR free interval > 16 months were correlated with improved mPFS (respectively 7.0 vs. 3.0 months, HR: 0.27, *p* = 0.013 and not reached vs. 3.0 months, HR: 0.20, *p* = 0.002) and with mOS (respectively 13.0 vs. 5.0 months, HR: 0.27, *p* = 0.013 and 13.0 vs. 5.0 months, HR: 0.20, *p* = 0.002). Previous lines >2 for metastatic disease were correlated with improved mPFS (4.0 vs. 1.0, HR: 0.05, *p* = 0.041) and mOS (7.0 vs. 1.0 months, HR: 0.045 *p* = 0.034). The optimal anti-EGFR free interval threshold identified for predicting prognosis was 14 months (**Figure 1**), whereas the optimal anti-EGFR free interval threshold identified for predicting response was 16 months (**Figure 2**). Prior responders with longer anti-EGFR free interval experienced the best clinical outcome in terms of RR, mPFS, and mOS (**Table 2**) to rechallenge strategy in our clinical series (**Figure 3**). 

**Table 2:** Clinical outcome results according to several clinical factors.

	RR	*p*	PFS (months)	HR	*p*	OS (months)	HR	*p*
**Anti-EGFR free interval 14 months** • >14 months • ≤14 months	36.4% 13.3%	0.347	7.0 (95% CI: 4.0–9.0) 3.0 (95% CI: 1.0–3.0)	0.27	0.013	13.0 (95% CI: 4.0–13.0) 5.0 (95% CI: 2.0–21.0)	0.28	0.019
**Anti-EGFR free interval 16 months** • >16 months • ≤16 months	44.4% 11.8%	0.137	not reached 3.0 (95% 95% CI: 2.0–9.0)	0.20	0.002	13.0 (95% CI: 3.0–13.0) 5.0 (95% CI: 3.0–21.0)	0.35	0.042
**Previous response to anti-EGFR** • **prior responders** • **prior non responders**	25.0% 16.7%	1.000	5.0 (95% CI: 3.0–9.0) 2.0 (95% CI: 1.0–9.0)	0.26	0.048	7.0 (95% CI: 5.0–13.0) 5.0 (95% CI: 2.0–21.0)	0.57	0.36
**Prior responders/Anti-EGFR free interval > 14 months** • yes • no	44.4% 11.8%	0.137	3.0 (95% CI: 2.0–9.0) not reached	0.21	0.0035	not reached 5.0 (95% CI: 3.0–21.0)	0.28	0.014
**Prior responders/Anti-EGFR free interval > 16 months** • yes • no	57.1% 10.5%	0.027	not reached 3.0 (95% CI: 2.0–9.0)	0.14	0.0003	not reached 5.0 (95% CI: 3.0–21.0)	0.30	0.026
**Oligometastatic disease lung limited** • **yes** • **no**	60.0% 14.3%	0.062	4.0 (95% CI: 2.0–4.0) 4.0 (95% CI:3.0–7.0)	1.61	0.420	12.0 (95% CI: 4.0–21.0) 5.0 (95% CI: 2.0–13.0)	0.38	0.120
**Oligometastatic disease liver limited** • **yes** • **no**	20% 23.8%	1.00	4.0 (95% CI: 1.0–7.0) 4.0 (95% CI: 3.00–9.00)	1.36	0.638	5.0 (95% CI: 2.0–13.0) 7.0 (95% CI: 4.0–21.0)	1.21	0.759
**Previous lines for metastatic disease** • **2** • **>2**	0.0% 25.0%	1.00	1.0 (95% CI: 1.0–3.0) 4.0 (95% CI: 3.0–9.0)	0.05	0.041	1.0 (95% CI: 1.0–5.0) 7.0 (95% CI: 5.0–21.0)	0.04	0.034
**Rechallenge regimen** • **Irinotecan + cetuximab** • **Cetuximab**	26.3% 14.3%	0.52	6.0 (95% CI: 4.6.0–8.2) 4.0 (95% CI: 4.1.0–7.2)	0.12	0.50	6.0 (95% CI: 6.7–14.8) 7.0 (95% CI: 4.3–12.9)	0.9	0.97

Response rate, progression-free survival, and overall survival according to anti-EGFR free interval > 14 months, anti-EGFR free interval > 16 months, previous response to anti-EGFR, prior responders with anti-EGFR free interval > 14 months, prior responders/anti-EGFR free interval > 16 months, oligometastatic disease (lung limited or liver limited), and previous lines for metastatic disease.

In a multiple logistic regression model, the anti-EGFR free interval >16 months as well as the anti-EGFR free interval >14, among the variables (i.e., long anti-EGFR free interval, previous response to anti-EGFR treatment, and previous lines >2 for metastatic disease), maintained their independent role for OS (*p* = 0.033 and *p* = 0.028, respectively). Likewise, the anti-EGFR free interval >16 months maintained the independent role for PFS (*p* = 0.020) among the significant variables in the univariate analysis (i.e., long anti-EGFR free interval, previous response to anti-EGFR treatment, and previous lines >2 for metastatic disease). None of them were statistically correlated to RR at multivariate analysis.

## Discussion

The rechallenge strategy with anti-EGFR antibodies can boast a plausible biological rationale along with promising results both from retrospective (6, 12, 13) and from phase II studies (7–9). However, prognostic and predictive factors, both molecular and clinical, along with the most effective treatment’s timeline are not yet fully defined. The response to previous anti-EGFR blockade and the anti-EGFR free interval are among the most studied prognostic and predictive factors for clinical-based rechallenge strategy. The genetic selection through ct-DNA analysis for RAS status, however, represents the most important tool in guiding patient selection, and the liquid biopsy-driven rechallenge with anti-EGFR showed improved outcome compared to clinical-based rechallenge strategy (9). Though the design of phase II trials investigating the rechallenge with anti-EGFR suggests a role in a third-line setting, a number of previous lines >2 (12) and longer anti-EGFR free interval (7, 8) were associated with improved clinical outcome. This suggests a possible usage in later lines (i.e., >3). Moreover, in clinical practice, rechallenge with anti-EGFR blockade is often used in later lines, whereas in third- and fourth-line settings, regorafenib and trifluridine-tipiracil are the preferred options on the basis of the phase III clinical trial data.

In the present study, we explored the rechallenge strategy with cetuximab-based therapy in clinically and molecularly selected, heavily pretreated (i.e., 92.3% received rechallenge with anti-EGFR in fourth- or fifth-line therapy) colorectal cancer patients. Notably, the response rate and the mPFS reported in the heavily pretreated population (i.e., RR: 25.0%, mPFS: 3.5 months) (**Table 1**) is worth mentioning and consistent with already available literature data from phase II studies.

Previous response to anti-EGFR, long anti-EGFR free interval, and previous lines >2 were correlated to better clinical outcome in our clinical series. In particular, previous responders with longer anti-EGFR free interval (i.e., >16 months) achieved the best outcome from rechallenge with anti-EGFR. PFS and OS were significantly longer in patients with long anti-EGFR free interval, consistent with results from phase II trials of clinical-driven rechallenge with anti-EGFR (7, 8). A number of previous lines >2 were associated with improved outcome (i.e., in terms of PFS and OS) according to results from previous retrospective analysis (i.e., improved outcome in terms of RR but no longer PFS and OS) (12). Similarly, the association of previous response to anti-EGFR with improved PFS is consistent with previous retrospective studies (13). Nevertheless, only long anti-EGFR free interval was significantly correlated to better outcome at multivariate analysis.

All this, in the light of the exponential decay kinetics of RAS mutant alleles after EGFR blockade suspension (5), suggests that the choice of the optimal timing of rechallenge strategy represents a key factor for this treatment approach and also that several factors others than RAS and BRAF mutations might be involved in secondary resistance to EGFR blockade (e.g., EGFR mutations).

Nevertheless, the results of the CHRONOS study (9) are partially contrary to our findings and to results from clinical-based rechallenge studies. In the CHRONOS study, the response occurred independently from the number of prior treatments, and the presence of resistance conferring mutations and responses was independent of time since last anti-EGFR. The very restrictive molecular inclusion criteria of this trial could possibly justify these findings, underlying the idea that the molecular selection is more effective in interpreting the dynamic nature of tumor biology compared to clinical factors.

Our study has some limitations; in particular, the small sample size along with the retrospective nature of the study deeply influenced the interpretations of our findings. This analysis, therefore, must be considered exploratory and caution is needed in data interpretation.

## Conclusions

Taking into account the current data regarding secondary resistance to EGFR blockade and the biological rationale for rechallenge strategy, the results of the present study are impressive and promising for future development. This especially applies in the form in which the results underlying the hypothesis of delaying the introduction of rechallenge with anti-EGFR would maintain efficacy in later lines. The liquid biopsy-driven anti-EGFR rechallenge is confirmed to be viable in clinical practice, and it should be considered the main tool for selected patients. Previous response to anti-EGFR and EGFR free interval might just represent a surrogate factor substituting in fact for acquired gene alteration status. Nevertheless, given the difficulties in applying the molecular methods from interventional liquid biopsy-driven anti-EGFR rechallenge studies, these clinical predictive factors maintain their value and a combination of the two approaches is also possible in the interest of greater effectiveness.

The up-to-date results from prospective liquid biopsy-driven rechallenge strategy trials (9, 14) are expected to give new insight into this challenging issue.

## Data Availability Statement

The raw data supporting the conclusions of this article will be made available by the authors upon request.

## Ethics Statement

The studies involving human participants were reviewed and approved by AOU Cagliari Ethical committee (approval number: 3.32 n.14 28/04/21). Written informed consent for participation was not required for this study in accordance with the national legislation and the institutional requirements.

## Author Contributions

All authors listed have made a substantial, direct, and intellectual contribution to the work and approved it for publication.

## Conflict of Interest

Author GiuP reports advisory role for BMS, MSD, Roche, Pierre-Fabre, Novartis, and Incyte.

The remaining authors declare that the research was conducted in the absence of any commercial or financial relationships that could be construed as a potential conflict of interest.

## Publisher’s Note

All claims expressed in this article are solely those of the authors and do not necessarily represent those of their affiliated organizations, or those of the publisher, the editors and the reviewers. Any product that may be evaluated in this article, or claim that may be made by its manufacturer, is not guaranteed or endorsed by the publisher.

## References

[1] Puzzoni M Ziranu P Demurtas L Lai E Mariani S Liscia N. Why Precision Medicine Should Be Applied Across the Continuum of Care for Metastatic Colorectal Cancer Patients. Future Oncol (2020) 16(2):4337–9. doi: 10.2217/fon-2019-0624 31793396

[2] Diaz LA Jr Williams RT Wu J Kinde I Hecht JR Berlin J. The Molecular Evolution of Acquired Resistance to Targeted EGFR Blockade in Colorectal Cancers. Nature (2012) 486(7404):537–40. doi: 10.1038/nature11219 PMC3436069 22722843

[3] Arena S Bellosillo B Siravegna G Martinez A Canadas I Lazzari L. Emergence of Multiple EGFR Extracellular Mutations During Cetuximab Treatment in Colorectal Cancer. Clin Cancer Res (2015) 21(9):2157–66. doi: 10.1158/1078-0432.CCR-14-2821 25623215

[4] Siravegna G Mussolin B Buscarino M Corti G Cassingena A Crisafulli G. Clonal Evolution and Resistance to EGFR Blockade in the Blood of Colorectal Cancer Patients. Nat Med (2015) 21(7):795–801. doi: 10.1038/nm0715-827b 26030179 PMC4868598

[5] Parseghian CM Loree JM Morris VK Liu X Clifton KK Napolitano S. Anti-EGFR-Resistant Clones Decay Exponentially After Progression: Implications for Anti-EGFR Re-Challenge. Ann Oncol (2019) 30(2):243–9. doi: 10.1093/annonc/mdy509 PMC6657008 30462160

[6] Santini D Vincenzi B Addeo R Carufi C Masi G Scartozzi M. Cetuximab Rechallenge In Metastatic Colorectal Cancer Patients: How to Come Away from Acquired Resistance? Ann Oncol (2017) 28(11):2906. doi: 10.1093/annonc/mdr623 28327895

[7] Cremolini C Rossini D Dell'Aquila E Leonardi S Conca E Del Re M. Rechallenge for Patients With RAS and BRAF Wild-Type Metastatic Colorectal Cancer With Acquired Resistance to First-line Cetuximab and Irinotecan: A Phase 2 Single-Arm Clinical Trial. JAMA Oncol (2019) 5(3):343–50. doi: 10.1001/jamaoncol.2018.5080 PMC6439839 30476968

[8] Masuishi T Tsuji A Kotaka M Nakamur M Kochi M Takagene A. Phase 2 Study of Irinotecan Plus Cetuximab Rechallenge as Third-Line Treatment in Kras Wild-Type Metastatic Colorectal Cancer: JACCRO CC-08. Br J Cancer (2020) 123(10):1490–5. doi: 10.1038/s41416-020-01042-w PMC7652864 32863385

[9] Sartore-Bianchi A Pietrantonio F Lonardi S Mussolin B Rua F Fenocchio E. Phase II Study of Anti-EGFR Rechallenge Therapy with Panitumumab Driven by Circulating Tumor DNA Molecular Selection in Metastatic Colorectal Cancer: The CHRONOS Trial. J Clin Oncol (2021) 39(15_suppl):3506. doi: 10.1200/JCO.2021.39.15_suppl.3506 34270348

[10] Mayer RJ Van Cutsem E Falcone A Yoshino T Garcia’Carbonero R Muzunuma N. Randomized Trial of TAS-102 for Refractory Metastatic Colorectal Cancer. N Engl J Med (2015) 372(20):1909–19. doi: 10.1056/NEJMoa1414325 25970050

[11] Grothey A Van Cutsem E Sobrero A Siena S Falcona A Ychou M. Regorafenib Monotherapy for Previously Treated Metastatic Colorectal Cancer (CORRECT): An International, Multicentre, Randomised, Placebo-Controlled, Phase 3 Trial. Lancet (2013) 381(9863):303–12. doi: 10.1016/S0140-6736(12)61900-X 23177514

[12] Rossini D Germani MM Pagani F Pellino A Dell’Aquila E Bensi M. Retreatment With Anti-EGFR Antibodies in Metastatic Colorectal Cancer Patients: A Multi-institutional Analysis. Clin Colorectal Cancer (2020) 19(3):191–9. doi: 10.1016/j.clcc.2020.03.009 32466976

[13] Liu X George GC Tsimberidou AM Naing A Wheler JJ Kopetz S. Retreatment with Anti-EGFR Based Therapies in Metastatic Colorectal Cancer: Impact of Intervening Time Interval and Prior Anti-EGFR Response. BMC Cancer (2015) 15:713. doi: doi.org/10.1186/s12885-015-1701-3 26474549 10.1186/s12885-015-1701-3 PMC4609167

[14] Metastatic Colorectal Cancer (RAS-wildtype) After Response to First-line Treatment With FOLFIRI Plus Cetuximab (AIO-KRK-0114). ClinicalTrials.gov Identifier. NCT02934529.

