# Supplementary figures and images for: Liquid Biopsy-Driven Cetuximab Rechallenge Strategy in Molecularly Selected Metastatic Colorectal Cancer Patients
Source: Front Oncol. 2022 Apr 21;12:852583. doi: 10.3389/fonc.2022.852583 (PMC9068964; doi:10.3389/fonc.2022.852583)

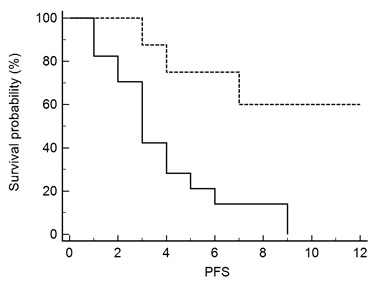

Supplement: Supplementary file 1 [file Image_1.jpeg]

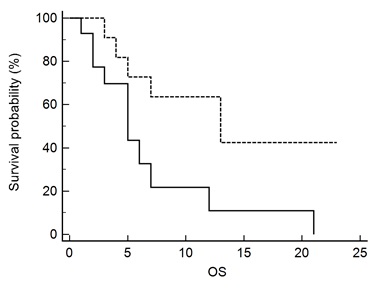

Supplement: Supplementary file 2 [file Image_2.jpeg]

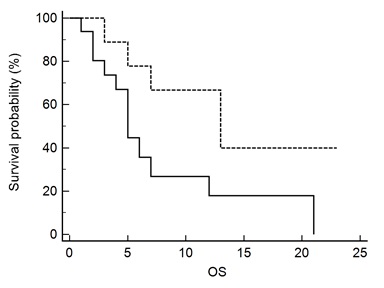

Supplement: Supplementary file 3 [file Image_3.jpeg]

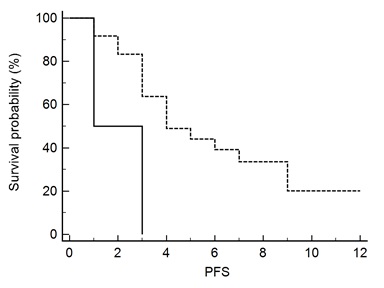

Supplement: Supplementary file 4 [file Image_4.jpeg]

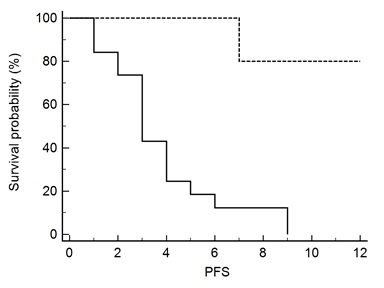

Supplement: Supplementary file 5 [file Image_5.jpeg]

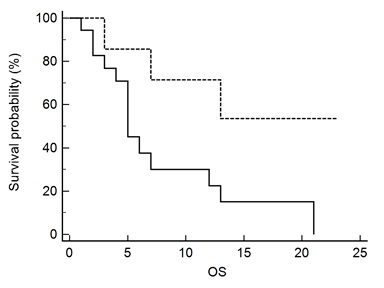

Supplement: Supplementary file 6 [file Image_6.jpeg]

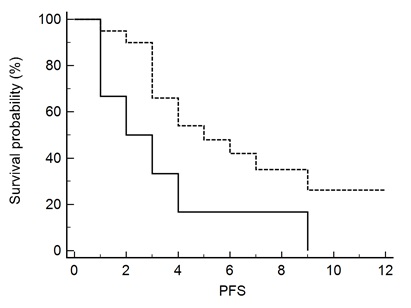

Supplement: Supplementary file 7 [file Image_7.jpeg]
